# AS03-adjuvanted H5N1 vaccine promotes antibody diversity and affinity maturation, NAI titers, cross-clade H5N1 neutralization, but not H1N1 cross-subtype neutralization

**DOI:** 10.1038/s41541-018-0076-2

**Published:** 2018-10-01

**Authors:** Surender Khurana, Elizabeth M. Coyle, Jody Manischewitz, Lisa R. King, Jin Gao, Ronald N. Germain, Pamela L. Schwartzberg, John S. Tsang, Hana Golding, and the CHI Consortium, Angelique Biancotto, Julián Candia, Jinguo Chen, Foo Cheung, Howard Dickler, Yuri Kotliarov, Shira Perl, Rongye Shi, Katherine E. R. Stagliano, Neal S. Young, Huizhi Zhou

**Affiliations:** 1 Division of Viral Products, Center for Biologics Evaluation and Research (CBER), FDA, Silver Spring, MD 20993 USA; 2 Center for Human Immunology (CHI), NIH, Bethesda, MD 20892 USA; 3 AstraZeneca, Gaithersburg, MD 20878 USA; 4 Department of Transfusion Medicine, NIH, Bethesda, MD 20892 USA

## Abstract

Immune responses to inactivated vaccines against avian influenza are poor due in part to lack of immune memory. Adjuvants significantly increased virus neutralizing titers. We performed comprehensive analyses of polyclonal antibody responses following FDA-approved adjuvanted H5N1-A/Indonesia vaccine, administered in presence or absence of AS03. Using Whole Genome Fragment Phage Display Libraries, we observed that AS03 induced antibody epitope diversity to viral hemagglutinin (HA) and neuraminidase compared with unadjuvanted vaccine. Furthermore, AS03 promoted significant antibody affinity maturation to properly folded H5-HA1 (but not to HA2) domain, which correlated with neutralization titers against both vaccine and heterologous H5N1 strains. However, no increase in heterosubtypic cross-neutralization of Group1-H1N1 seasonal strains was observed. AS03-H5N1 vaccine also induced higher neuraminidase inhibition antibody titers. This study provides insight into the differential impacts of AS03 adjuvant on H5N1 vaccine-induced antibody responses that may help optimize vaccine platforms for future vaccines with improved protection against seasonal and pandemic influenza strains.

## Influenza: understanding the mechanisms of vaccine-boosting additives

Adjuvant AS03 improves a bird flu vaccine’s ability to recognize and bind to virion targets. Avian influenza viruses are considered a pandemic threat, and vaccines made from inactivated virus are limited in their efficacy. However, adjuvants such as AS03 have the ability to augment a host’s immunity, prompting Surender Khurana and colleagues from the United States’ Food and Drug Administration to investigate how. In an H5N1 vaccine trial, researchers found that AS03 improved immune responses by increasing the degree, diversity, and intensity of antibody binding to viral surface proteins, which likely caused the observed increase in the neutralization of the virus tested as well as efficacy against other H5N1 strains. The AS03-adjuvanted vaccine did not neutralize seasonal H1N1 influenza strains. This study may inform future vaccine development efforts by partially illuminating the effects of AS03.

## Introduction

Humans are mostly immune-naïve to avian influenza strains as they have not been exposed to these strains in their lifetime. Transmission of avian strains from wild birds to poultry with subsequent transmission to humans resulted in high morbidity and mortality among infected individuals and represents pandemic threat. Highly pathogenic (HP) H5N1 viruses still cause significant lethality in bird populations with numerous instances of human transmission resulting in severe human disease with >60% mortality.^1,2^ As of May 28, 2018, there were 860 confirmed human cases with 454 fatalities. (http://www.who.int/influenza/human_animal_interface/2018_05_28_tableH5N1.pdf?ua=1). Furthermore, low pathogenic avian influenza (LPAI) H7N9 viruses have caused five epidemic waves of human infections in China since 2013, and the number of human infections increased significantly during the fifth wave. As of July 26, 2017, there were 1582 laboratory-confirmed human cases of avian H7N9 infection, with 39% fatality rate. In February 2017, highly pathogenic H7N9 viruses possessing multibasic cleavage site in the hemagglutinin (HA) were detected in China.^3–5^ Therefore, both avian-derived A/H5N1 and A/H7N9 influenza viruses are considered a pandemic threat due to lack of preexisting immunity in humans. However, inactivated influenza vaccines containing HA and neuraminidase genes from avian influenza reassorted with the A/Puerto Rico/8/34 (PR8) internal genes were found to be poorly immunogenic in human trials, even when the HA content was increased significantly compared with seasonal influenza vaccines.^6,7^

Adjuvants comprised of diverse immune modulators that have been used with vaccines to boost the immune responses by various mechanisms, for example, through upregulating genes in antigen-presenting cells that migrate from the site of vaccination to the draining lymph nodes and promote activation of Th, Tfh, and B cells and cytokine production.^8,9^ Oil in water adjuvants MF59 and AS03 have been evaluated in multiple preclinical and clinical studies in combination with several influenza vaccines (both seasonal and avian influenza strains), and were found to significantly augment the humoral immune responses as determined by hemagglutination inhibition (HAI) and microneutralization (MN) assays.^10–13^

One of the desired attributes of vaccines against avian influenza strains with pandemic potential is the ability to elicit long-term broadly reactive antibodies that could afford at least partial protection against the emerging pandemic strains. Influenza vaccines against avian influenza when combined with oil-in-water adjuvants (MF59 and AS03) generated cross-clade HAI and MN titers and demonstrated improved cross-reactive titers after heterologous prime-boost regiments.^12,14,15^ With the continuous introduction of HP avian influenza strains to humans, rapid evolution and diversification of the H5N1 and H7N9 viruses have highlighted the need for advance stockpiling of H5N1 and H7N9 vaccines, anticipating that vaccination with the stockpiled vaccine can provide at least partial protection until a vaccine matching the pandemic strain becomes available for mass vaccination.^16–19^ In addition, stockpiled vaccine could be served to prime recipients for later pandemic-specific vaccination with a single vaccine dose. The AS03-adjuvanted H5N1 A/Indonesia/05/2005 subunit vaccine was approved by the FDA in 2013 and is currently in the US National stockpile.

To better understand the adjuvant impact on the humoral immune response to influenza vaccines, we developed several methodologies including whole-genome-phage-display libraries (GFPDL) to probe the epitope repertoires of antibodies elicited in humans by adjuvanted vs. unadjuvanted vaccines. In addition, properly folded HA1 and HA2 domains were used to measure total binding and the dissociation rates of the bound polyclonal antibodies in human polyclonal plasma/serum as correlates of antibody affinity.^20–24^

Since current influenza virus vaccines have demonstrated limited clinical efficacy in recent years, there is an urgent need for next-generation vaccines that can provide broader, long-lasting protection against influenza A and B viruses. It is also expected that adjuvants will be important in the development of the next-generation improved influenza vaccines (termed “universal”), that aim to provide cross-subtypic heterologous protection against diverse influenza strains.

In the current study, we performed a comprehensive analysis of the antibodies elicited by the FDA-approved AS03-adjuvanted H5N1 (A/Indonesia/05/2005) vaccine by: (1) measuring the diversity of antibody epitope repertoires in HA and NA, (2) evaluating the binding affinity to HA1 vs. HA2 domains, and (3) correlating these results with functional assays (MN and neuraminidase inhibition assay) against homologous and heterologous H5N1 strains. To explore the possibility of oil-in-water adjuvants to augment cross-subtype neutralizing antibodies, we also evaluated the capacity of the AS03-adjuvanted H5N1 vaccine to elicit heterosubtypic (stem-targeting) neutralizing antibodies against H1N1 group 1 virus strains that circulated in the previous decade.

## Results

### AS03 improves immune response against homologous and heterologous avian influenza strains

The NIH/Center for Human Immunology (CHI) clinical study vaccinated healthy adult volunteers with FDA-licensed monovalent Influenza H5N1 Virus Vaccine (A/Indonesia/05/2005) formulated in combination with or without AS03 adjuvant.

**Fig. 1:**
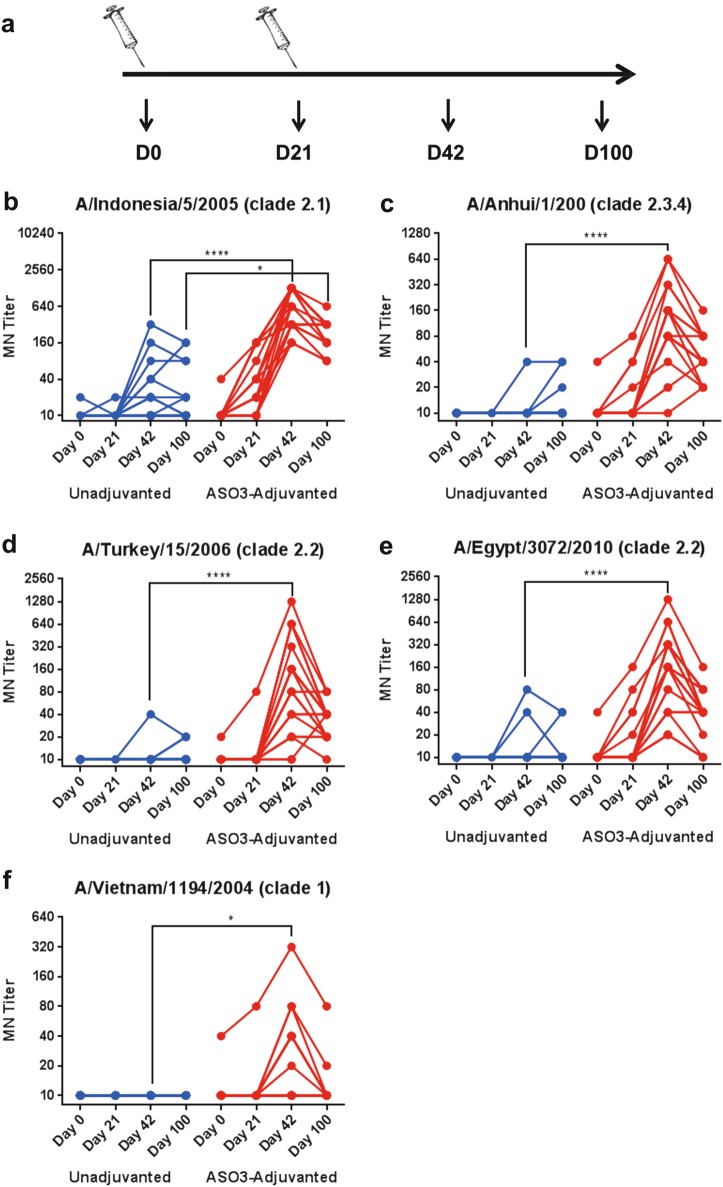
Endpoint microneutralization titers following vaccination of adults with H5N1 with and without AS03 adjuvant against diverse H5N1 strains. **a** Schematic design of the heterologous H5N1 prime-boost vaccine immunization is shown. The number of individuals in each group: Unadjuvanted = 20; AS03-adjuvanted = 22. **b−f** End-point microneutralization titers of the individuals against various H5N1 virus strains at day 0 (prevaccination), day 21 (post first vaccination), day 42 (post second vaccination) and day 100 postvaccination are shown for AS03-adjuvanted group (in red) and unadjuvanted group (in blue) against **b** homologous H5N1 A/Indonesia/5/2005 (clade 2.1) vaccine strain, **c** A/Anhui/1/2005 (clade 2.3.4). **d** A/Turkey/15/2006 (clade 2.2), **e** A/Egypt/3072/2010 (clade 2.2), and **f** A/Vietnam/1194/2004 (clade 1). The pairwise comparison of endpoint neutralization titers that were statistically significant with *p* values of <0.05 (*), or <0.005 (**), or <0.0005 (***), or <0.0001 (^****^) are shown

We explored the impact of adjuvant on the quality of the antibody responses against the H5N1 vaccine strain as well as heterologous influenza strains. All subjects were vaccinated twice (on days 0 and 21) with H5N1 A/Indonesia/05/2005 monovalent inactivated vaccine (GSK), containing 3.75 µg HA, either alone or mixed (1:1) with AS03 (oil-in-water) adjuvant (Fig. 1a). Serum samples from blood drawn on days 0, 21, 42, and 100 (Fig. 1a) were used in MN assay of diverse H5N1 influenza strains using MDCK cells. In the unadjuvanted vaccine group the seroconversion rate (SCR; % of individuals showing greater than or equal to fourfold rise from baseline in MN and reaching titers ≥1:40) against the H5N1-A/Indonesia vaccine strain was 0% after the first vaccination (day 21) and reached only 25% on day 42 (21 days post second boost), respectively (Table 1 and Fig. 1b, blue symbols). In contrast, the SCR in the AS03-adjuvanted vaccine group was 45% on day 21 (3 weeks after first immunization), and reached 100% on day 42 (Table 1 and Fig. 1b, red symbols). Most importantly, the geometric mean neutralization titers measured in serum from the AS03-adjuvanted vaccine group were 30-fold higher compared with the unadjuvanted vaccine group by day 42 (Table 1). By day 100, the % of subjects with MN titers above 1:40 was unaltered in the AS03-adjuvanted vaccine group (100%), but the GMT values dropped threefold compared to day 42 (Table 1 and Fig. 1b). We also looked at gender differences. Slightly higher neutralization titers against the vaccine strain were found on day 42 (3 weeks post second vaccination) in females compared to males (*p* < 0.05), but no differences were observed either earlier (day 21) or later (day 100) time-point postvaccination (Supplementary figure 1).

**Table 1:** Microneutralization endpoint titers for H5N1 virus strains

			A/Indonesia/5/05 (Clade 2.1)			A/Turkey/15/2006 (Clade 2.2)			A/Egypt/3072/2010 (Clade 2.2)			A/Anhui/1/2005 (Clade 2.3.4)			A/Vietnam/1203/2004 (Clade 1)		
Group	Vaccine	Day	SCR (%)^a^	GMT	SD	SCR (%)^a^	GMT	SD	SCR (%)^a^	GMT	SD	SCR (%)^a^	GMT	SD	SCR (%)^a^	GMT	SD
1	Unadjuvanted	0		10.35	2.24		10	0		10	0		10	0		10	0
	(*n* = 20)	21	0	10.35	2.24	0	10	0	0	10	0	0	10	0	0	10	0
		42	25	20	74.64	5	10.72	6.71	10	11.89	16.7	5	10.72	6.71	0	10	0
		100	21	18.59	49.14	0	11.16	3.75	11	11.57	9.46	11	12	9.55	0	10	0
2	AS03	0		10.65	6.4		10.32	2.13		10.65	6.4		10.65	6.4		10.65	6.4
	(*n* = 22)	21	45	31.09	60.53	5	10.99	15.95	18	14.59	34.83	14	12.87	16.77	5	10.99	14.92
		42	100	600.92	487.05	77	93.65	313.55	86	136.68	291.7	86	106.23	173.42	36	20	66.76
		100	100	218.57	133.05	75	34.82	20.12	80	40	35.02	80	45.95	33.78	5	11.49	15.69

a All subjects were seronegative on day 0. SCR, % of individuals showing greater than or equal to fourfold rise from baseline in MN and reaching titers > 1:40

The same serum samples were evaluated for H5N1 cross-clade neutralization. As can be seen in Table 1 and Fig. 1 (panels c−f, red symbols), significant cross-clade neutralization titers were evident from the AS03-adjuvanted vaccine group, reaching SCRs of 86, 77, 86, and 36%, against H5N1; A/Turkey/15/2006 (clade 2.2), A/Egypt/3072/2010 (clade 2.2), A/Anhui/1/2005 (clade 2.3.4), and A/Vietnam/1194/2004 (clade 1), respectively. However, the maximum GMT values were lower than against the vaccine strain (H5N1 A/Indonesia/05/2005; clade 2.1). In contrast, cross-clade neutralization titers were extremely low or undetectable for the unadjuvanted H5N1 vaccine group (Table 1 and Fig. 1, panels c−f, blue symbols).

### H5N1 gene-fragment-phage-display-library (FLU-GFPDL) analysis demonstrates epitope repertoire expansion of polyclonal antibodies to HA and NA in AS03-adjuvanted H5N1 vaccine recipients

**Fig. 2:**
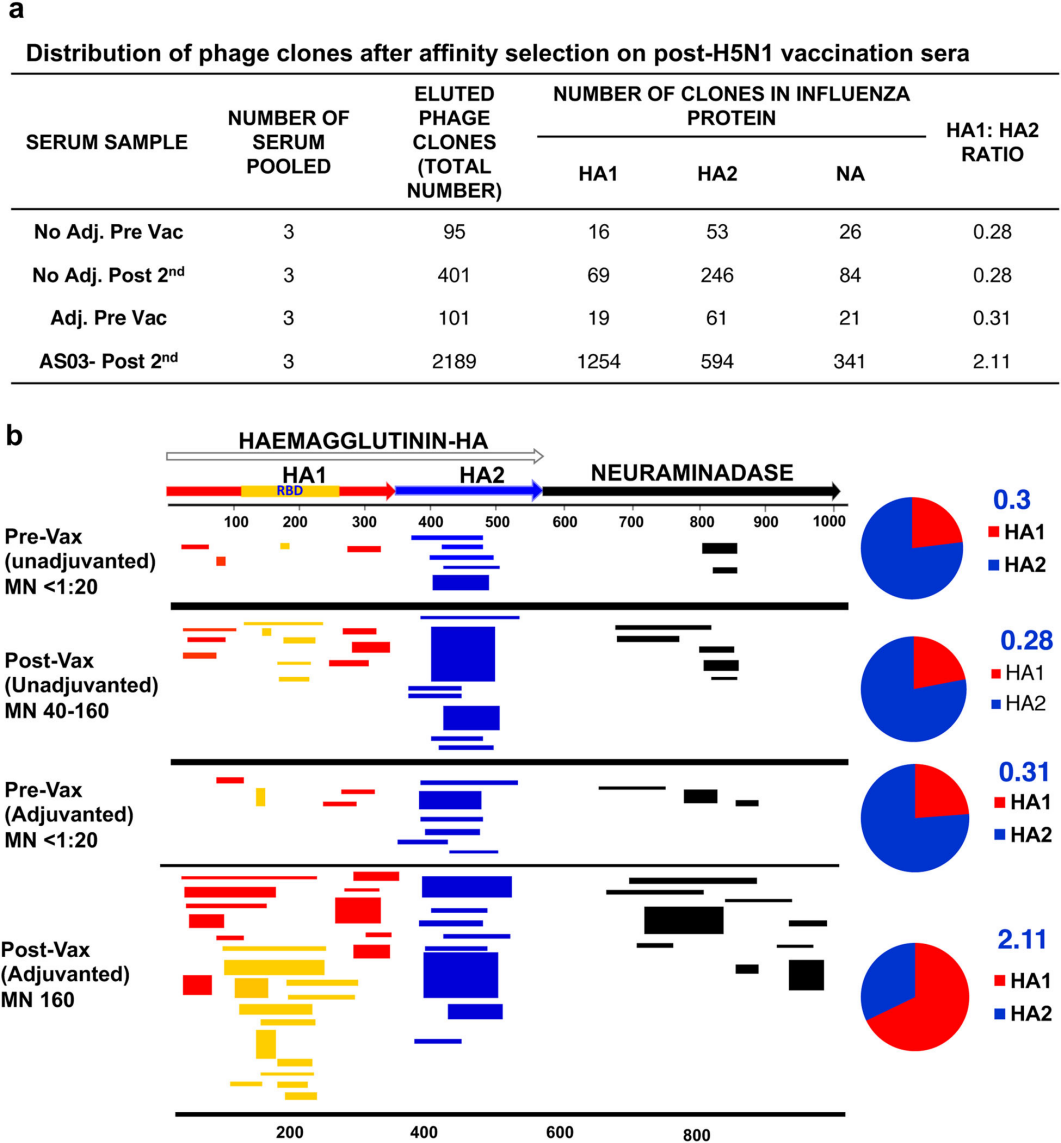
Elucidation of antibody repertoires elicited in humans following vaccination with unadjuvanted and adjuvanted subunit H5N1-A/Indonesia vaccine. **a** Distribution of phage clones after affinity selection on post-H5N1 vaccination sera. **b** Schematic alignment of the peptides recognized by post-second vaccination sera in the NIAID sponsored trial, identified by panning with H5N1-GFPDL A/Indonesia/5/2005. Amino acid designation is based on the HA + NA protein sequence (Fig. S1). Bars with arrows indicate identified inserts in the 5′-3′ orientation in HA1 (red bars), HA2 (blue bars), and NA (black bars). Only clones with frequency of ≥2 are shown. Yellow bars indicate inserts spanning the receptor binding domain (RBD) in HA1. The pie charts represent the ratio of frequency of clones selected in HA1 (red) vs. HA2 (blue) domain by various vaccine groups are depicted. The thickness of bars represents the frequency of phages containing each insert. All bars shown represent phages that were affinity selected at least twice

To further evaluate the impact of the AS03 adjuvant on the quality of the antibody response to H5N1 vaccine, serum samples from a subset of seroconverted subjects enrolled in the NIH/CHI study were pooled at prevaccination and at day 42 (3 weeks after the second vaccination). All prevaccination samples were seronegative (MN titers < 1:20). For the postvaccination serum pooling, samples that had similar MN titers in the unadjuvanted (MN titers: 40−160) and adjuvanted vaccine (MN titers: 160) groups were selected to explore qualitative differences in antibody epitope repertoires following vaccination. Affinity selection was performed on samples using the FLU-GFPDL constructed from HA and NA genes of H5N1 A/Indonesia/5/05 vaccine strain (Fig. 2), similar to as described previously for the A/Vietnam GFPDL.^20–22^

The numbers of bound phages by prevaccination serum antibodies were low (95, 101) in both vaccine groups (Fig. 2a). Immunization with the unadjuvanted vaccine resulted in a fourfold increase in the number of bound phages, while sera from AS03-adjuvanted vaccine recipients showed a >20-fold increase in the number of bound phages for day 42 serum samples (401 and 2189 bound phages, respectively) (Fig. 2a).

Mapping of the sequenced inserts from the bound phages is illustrated in Fig. 2b. Interestingly, prevaccination serum antibodies bound to H5 HA inserts that mapped primarily to a region in the HA2 stalk with very high sequence homology (98%) to the HA2 of seasonal H1N1 strains (blue lines, Fig. 2b top panel). As expected, binding to the HA1 globular head was minimal and did not encompass the receptor binding domain (RBD) (yellow lines, Fig. 2b top panel). Postvaccination pooled sera from the unadjuvanted vaccine group primarily reacted with phages expressing HA2 epitopes (61% of clones), with increase in total number of phages bound, but minimal increase in the diversity of HA2 inserts, suggesting a memory recall response (Fig. 2b, second panel). These serum antibodies also recognized some HA1 inserts that were relatively short, mapping to the RBD and the N and C-terminals of HA1, at low frequency (17% of total clones) (Fig. 2a, b, second panel). In contrast, pooled sera from individuals vaccinated with two doses of AS03-adjuvanted H5N1 vaccine displayed a diverse expanded antibody epitope profile. In addition to the tenfold increase in the number of HA2-specific antibodies targeting the conserved H1/H5 region, there was a pronounced epitope spreading of antibodies targeting sites in the HA1 domain (57% of total inserts). Importantly, only sera from the AS03-adjuvanted vaccine group recognized long HA1 sequences encompassing the RBD, which are expected to contain conformational epitopes, likely to be recognized by protective antibodies^20^ (Fig. 2a, b bottom panel). The ratios of HA1/HA2 bound inserts were 0.3 for the prevaccination samples from both vaccine groups as well as for the postvaccination sera from the unadjuvanted vaccine group, suggesting dominance of the stem-specific antibodies. In contrast, the HA1/HA2 ratio of bound phages was 2.1 for the pooled sera from the AS03-adjuvanted vaccine group (Fig. 2b, pie charts) reflecting the change in epitope dominance within the HA towards the HA1 globular head domain, in spite of the increase in HA2-targeting antibodies.

The postvaccination sera from the AS03-adjuvanted H5N1 vaccine group also bound to a larger number of inserts mapping to the H5N1 neuraminidase protein (16-fold increase) compared with prevaccination sera and fourfold higher than postvaccination samples from the unadjuvanted H5N1 vaccine group (Fig. 2a, b, black lines). The increase in bound inserts included new epitopes mapping to the C-terminus of NA, close to the sialic acid binding enzyme active site.^25^

These data demonstrated both strong recall responses and epitope spreading of antibody repertoire following vaccination with AS03-adjuvanted (but not unadjuvanted) H5N1 vaccine.

### AS03-adjuvanted H5N1 vaccine induces higher reactivity to properly folded HA1 globular head domain

Neutralization of influenza virus occurs primarily by antibodies recognizing conformational epitopes located on the globular head of the HA molecule^26,27^ surrounding the RBD. Previously, we demonstrated that sera from H5N1-infected convalescing individuals and human MAbs derived from H5N1 survivors bound to cross-clade conserved conformational epitopes presented on large HA1 fragments identified by panning on GFPDL from the H5N1-A/Vietnam strain.^20^

Therefore, it was important to measure the quality of antibodies elicited by the H5N1 vaccine administered with or without AS03 adjuvant, both in terms of titer and avidity of antibodies, using a surface plasmon resonance (SPR)-based real-time kinetics assay to properly folded HA1 globular domain. To that end, purified H5 HA1 protein was produced and characterized for: (1) the presence of oligomers by gel filtration chromatography; (2) functional binding to sialic acid containing glycans in SPR, and (3) binding to red blood cells resulting in hemagglutination, as previously described.^23^

**Fig. 3:**
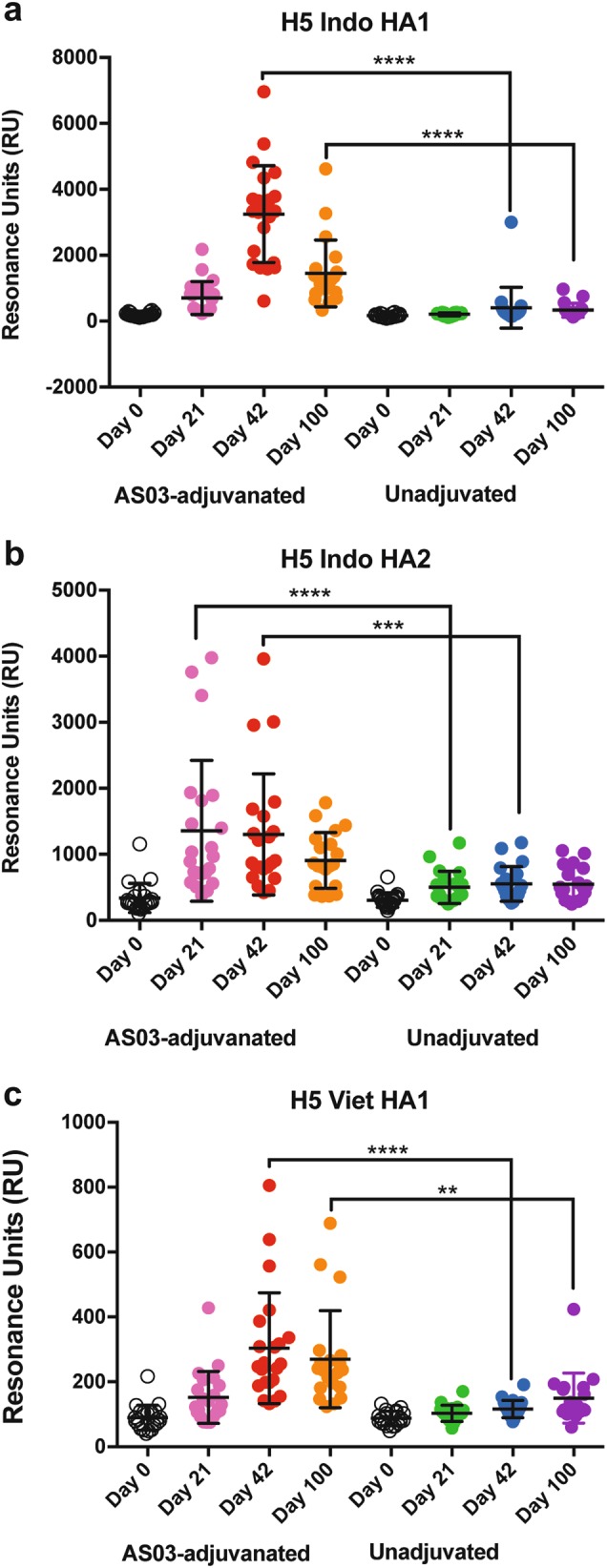
Binding of post-H5N1 vaccination human sera to properly folded HA1 and HA2 domains. **a**−**c** Steady-state equilibrium analysis of human vaccine sera at day 0 (prevaccination), day 21 (post first vaccination), day 42 (post second vaccination) and day 100 postvaccination are shown for AS03-adjuvanted group and unadjuvanted group against properly folded homologous H5-A/Indonesia/5/05 (Clade 2.1) HA1 (**a**) and HA2 (**b**) domains and heterologous H5N1-A/Vietnam/1203/2004 (Clade 1) HA1 domain (**c**) were measured using SPR. Recombinant HA1 and HA2 domains were immobilized on a sensor chip through the free amine group. Binding of the antibodies to the immobilized protein is shown as resonance unit (RU) values. Mean with standard deviation (SD) is also shown. The pairwise comparison of serum polyclonal antibody binding titers that were statistically significant with *p* values of <0.05 (*), or <0.005 (**), or <0.0005 (***), or <0.0001 (^****^) are shown

As can be seen in Fig. 3a, polyclonal antibody binding of individual serum samples from the AS03-adjuvanted H5N1 vaccine recipients to properly folded HA1 domain increased moderately by day 21 after the first vaccination. A significant increase in total anti-HA1 antibody binding was observed on day 42 (3 weeks post second vaccination). However, the postvaccination antibody binding titers (Max RU) were significantly reduced by day 100 (Fig. 3a). We also measured antibody binding to the HA1 domain from heterologous H5N1 A/Vietnam/1203/2004 (clade 1), and found a similar pattern of increase in binding, peaking on day 42, but much lower binding titers compared to homologous HA1 from A/Indonesia/5/05 (Fig. 3c). In contrast, sera from the unadjuvanted vaccine group showed minimal antibody binding to HA1 from A/Indonesia or A/Vietnam on day 21 and very low binding on D42 and D100 (Fig. 3a, c).

As can be seen in Fig. 3b, antibody binding to the H5-HA2 domain was elevated already by day 21, suggesting a memory recall response that was maintained on day 42, and only declined marginally by day 100. Anti-HA2 antibody binding was significantly higher for the AS03-adjuvanted vaccine group compared with the unadjuvanted vaccine sera on both days 21 and 42 (Fig. 3b).

For measurements of antibody affinity maturation, serum samples at 10-fold and 100-fold dilutions were injected on a low density chip surface so as to measure monovalent antibody−antigen interactions of serum antibodies in SPR. Antibody off-rate constants, which describe the fraction of antigen−antibody complexes that decay per second, were determined directly from the serum sample interaction with rHA1 or rHA2 protein using SPR in the dissociation phase as described before.^22^

**Fig. 4:**
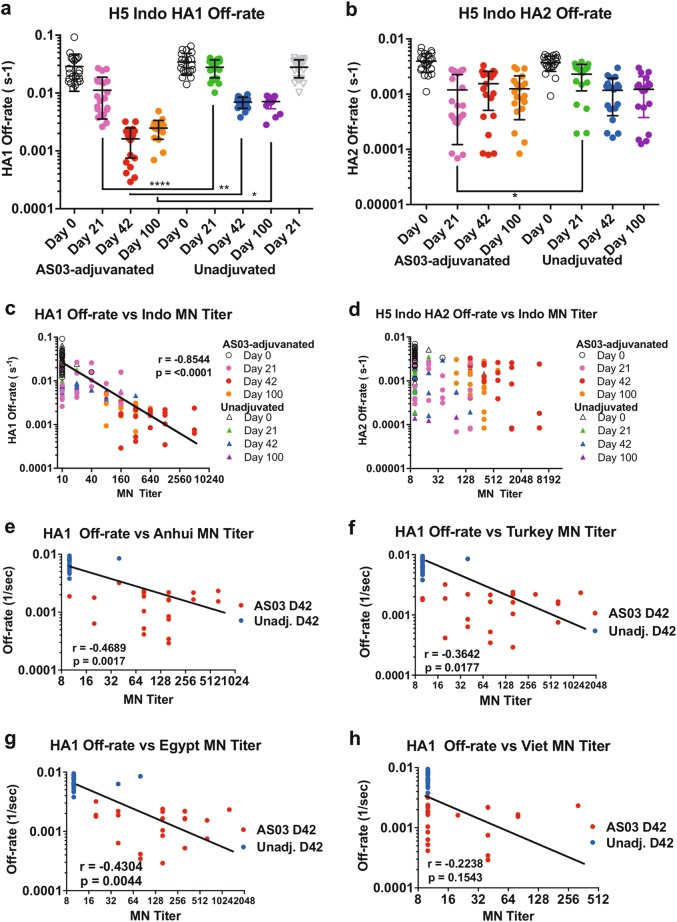
Kinetics of antibody affinity maturation following vaccination with adjuvanted and unadjuvanted H5N1 subunit vaccine. Sequential SPR analysis of vaccine sera (pre-, post first and post second vaccination with unadjuvanted or AS03-adjuvanted H5N1 vaccine) with properly folded homologous H5-A/Indonesia/5/05 HA1 (**a**) and HA2 (**b**) domains. Ten-fold and 100-fold diluted individual serum from each participant in vaccine trial at prevaccination (day 0) and at 21 days after each immunization, as well as at day 100 were evaluated. Serum antibody off-rate constants were determined as described in the Material and Methods. Mean with standard deviation (SD) is also shown. The pairwise comparison of polyclonal antibody off-rates that were statistically significant with *p* values of <0.05 (*), or <0.005 (**), or <0.0005 (***), or <0.0001 (^****^) are shown. **c**, **d** Correlation between in vitro neutralizing titers against homologous H5N1-A/Indonesia/5/05 and anti-HA1 antibody affinity (**c**) and anti-HA2 binding affinity (**d**) in human sera before and after H5N1 vaccination is shown. **e**−**h** Correlation statistics of the off-rate constants and peak MN titers on day 42 (post second vaccine) for heterologous H5N1 strains are shown and found to be highly significant for **e** A/Anhui/1/2005 (clade 2.3.4), **f** A/Turkey/15/2006 (clade 2.2), **g** A/Egypt/3072/2010 (clade 2.2), with *p* < 0.05 but not for highly divergent **h** A/Vietnam/1194/2004 (clade 1) virus

As can be seen in Fig. 4, the dissociation rates for antibodies from the AS03-adjuvanted vaccine group declined gradually after the first and second vaccination reflecting a ≥20-fold increase in antibody binding affinity on D42 compared with D0. Serum antibodies on day 100 demonstrated maintenance of high affinity binding antibodies to the HA1 domain in this vaccine group. In comparison, for the unadjuvanted vaccine samples, the increase in HA1-binding antibody affinity was limited (~5-fold) (Fig. 4a). The binding affinities to the HA2 stem were ~10-fold higher (10^−2^/sec) compared to HA1-binding antibodies (10^−1^/sec) on day 0 (prevaccination). Following vaccination, the affinities of anti-HA2 antibodies were highly variable within groups with no major increase after the second boost. The affinity of HA2 binding antibodies were also similar between the adjuvanted and unadjuvanted groups, except for significant difference observed on day 21 post first vaccination (Fig. 4b).

These data demonstrated that compared with the unadjuvanted vaccine, the AS03-adjuvanted H5N1 vaccine elicited higher affinity antibodies for the HA1 globular domain that persisted for 100 days even when the total HA1-binding antibody titers were reduced (compare Figs. 4a and 3a).

### Homologous and heterologous neutralization of polyclonal sera correlates with antibody affinity for the HA1 domain of H5 A/Indonesia

To determine the correlation between the MN titers of individual study participants (Fig. 1) and binding affinities to H5-HA1 and HA2 domains, Spearman correlations were determined for neutralization titers against the homologous vaccine strain of all study participants at all time points vs. antibody binding off-rates to the A/Indonesia HA1 and HA2 (Fig. 4c−h). Statistically significant inverse correlations were found between antibody off-rates to A/Indonesia HA1 and MN titers against A/Indonesia (vaccine strain) on all sample days. This inverse correlation was also observed for day 42 post vaccination A/Indonesia anti-HA1 antibody off-rates and MN titers against the other heterologous H5N1 strains representing different clade 2 lineages (but not clade 1), with *p* values ranging between 0.0017−0.0001 (Fig. 4c, e, f, g). No significant correlation was found between binding serum antibody affinities to the HA2 stem and neutralization titers against the vaccine strain or the heterologous H5N1 influenza strains (Fig. 4d and data not shown).

These data suggested that the AS03 adjuvant enhanced the recruitment of naïve B cells with specificity to the HA1 globular domain of H5N1 A/Indonesia and promoted significant affinity maturation that translated into high seroprotection rates up to 100 days postvaccination. The increase in anti-HA1 antibody binding affinity probably contributed to cross-clade H5N1 neutralization.

### Evaluation of heterotypic neutralization of Group 1 H1N1 seasonal strains following vaccination with AS03-adjuvanted H5N1 vaccine

Since the discovery of broadly neutralizing monoclonal antibodies targeting the conserved epitopes in the HA stem or the RBD of the influenza HA^28–31^ there is an accelerated effort to develop new vaccine modalities to generate sustained broader immune responses that could neutralize/protect against drifted seasonal strains and avian strains with pandemic potential.^32,33^

**Fig. 5:**
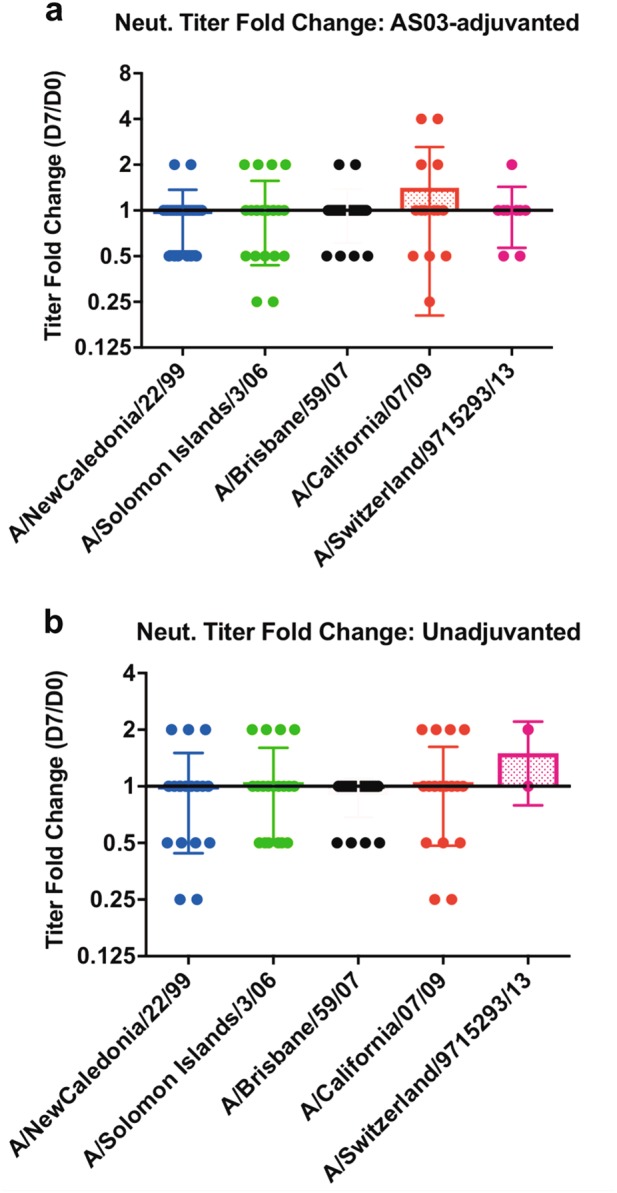
No increase in neutralization titers against Group 1 seasonal H1N1 strains following vaccination with AS03-adjuvanted H5N1 vaccine in adults. To ascertain the generation of stem-specific neutralizing antibodies that will cross neutralize Groups 1 H1N1 strains following vaccination with AS03-adjuvanted H5N1 vaccine, a modified microneutralization assay was performed (as described in the Materials and Methods) with serum samples from day 0 (prevaccination) and day 7 post first vaccination (demonstrated to have peak HA2 stem cross-reactive titers following heterologous H5N1 vaccination). No significantly higher fold change (day 7/day 0) in neutralization titers were observed for any of the Gp 1 H1N1 (A/New Caledonia/22/99, A/Solomon Islands/3/06, A/Brisbane/59/07, A/California/07/2009), or Gp 2 H3N2 (A/Switzerland/9715293/2013) viruses following vaccination AS03-adjuvanted H5N1 (**a**) or unadjuvanted H5N1 vaccine (**b**). Mean with standard deviation (SD) is also shown

We observed an increase in antibody binding to the HA2 stem domain in the sera from AS03-adjuvanted H5N1 vaccine recipients that did not correlate with the neutralization titers against H5N1 strains using the standard 1 day MN assay (CDC protocol). To further explore if the increase in HA2 stem binding antibodies following AS03-adjuvanted H5N1 vaccination led to any increase in stem-targeting neutralizing antibodies, we employed a modified MN assay (3-day) that was previously described by other investigators^34^ to measure neutralization of heterosubtypic Group 1 H1N1 viruses that circulated in the US in the previous decade. The strains used, H1N1 A/New Caledonia/22/99, A/ Solomon Islands/3/06, and Brisbane/59/07 circulated in the US in the years 2000–2007, 2007/8, and 2009/10, respectively. These strains preceded the emergence of the pandemic H1N1 A/California/04/09 in 2009 (antigenically similar to the 1918 pandemic strain) that replaced the previous H1N1 seasonal strain and continues to circulate in the world today. Most adults have very low or no antibody titers against the old H1N1 strains. Since the H5N1 vaccine strain has a novel head domain compared to H1N1 strains we expected participants in the study to have very low induction of head-specific H1N1 cross-reactive antibodies that can block receptor binding (captured by HAI or traditional MN assay) following H5N1 vaccination, yet be likely to have cross-reactive stem-specific memory B cells that could have been recalled by the AS03-adjuvanted H5N1 vaccine due to the homology between H5 and H1 HA2 stem domain as shown in Fig. 2. For this analysis, we used serum samples from day 0 (prevaccination) and day 7 (post first vaccination) as previously recommended for capturing memory plasmablasts against the HA2 stem domain.^35,36^ MAb CR6261 was used as a positive control in these assays. This stem-targeting MAb was reported to have broad in vitro neutralizing activity against Group 1 H1, H2, H5, H6, H8, H9 influenza subtypes.^28,30,37^ In the modified MN assay, CR6261 neutralized A/New Caledonia/22/09 and A/Solomon Islands/3/06 with endpoint of 0.025–0.05 mg/ml. However, neutralization of A/Brisbane/59/07 was much weaker (>0.1 mg/ml). In addition to the older H1N1 strains, we measured neutralization of H1N1 A/California/04/09, and Gp 2 H3N2 seasonal strain A/Switzerland/9715293/13 (Fig. 5).

The prevaccination (day 0) samples from study participants had low neutralizing antibody titers against these H1N1 seasonal influenza strains ranging between <1:10 and 1:160. Postvaccination (day 7) sera samples from both unadjuvanted and adjuvanted groups showed either reduction, no change, or minimal (twofold) increase in neutralization titers, with only two individual samples showing fourfold increases in titers over day 0 against pandemic H1N1 strain in the adjuvanted vaccine group (Fig. 5a, b). Therefore, in the modified neutralization assay, similar to the traditional MN assay, we did not find evidence for an increase in stem-targeting heterosubtypic neutralizing antibodies.

### AS03 adjuvant promotes increase in neuraminidase inhibition (NAI) titers following H5N1 vaccination

**Fig. 6:**
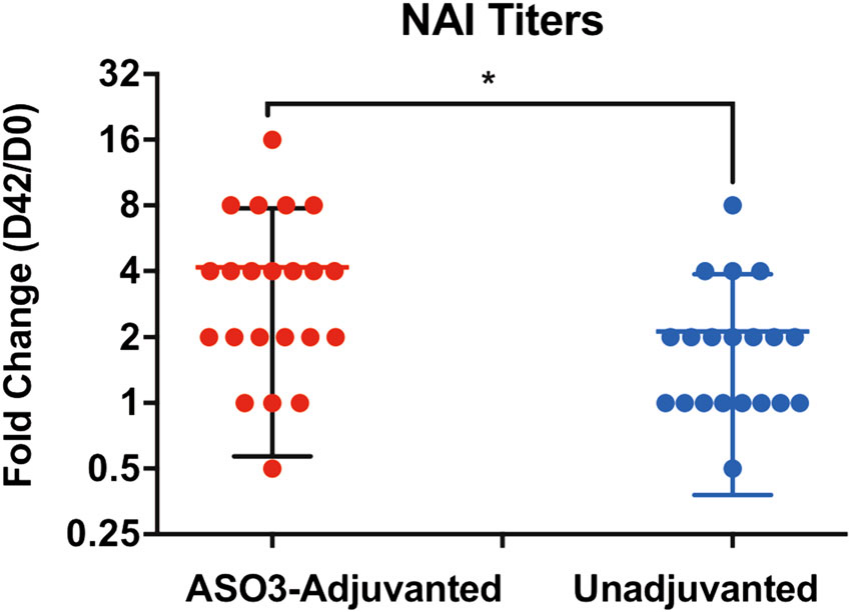
AS03 adjuvant promotes H5N1 neuraminidase-inhibiting antibodies. Neuraminidase enzymatic activity inhibition (NAI) titers for prevaccination (day 0) and post second vaccination (day 42) human sera were measured as described in the Material and Methods. The pairwise comparison of sera from AS03-adjuvanted vaccine recipients gave significantly higher fold change in NAI titers compared to unadjuvanted H5N1 vaccines with *p* values of <0.05, respectively. Mean with standard deviation (SD) is also shown

Current influenza inactivated vaccines contain varying levels of neuraminidase (NA) in addition to HA, but there is no regulatory requirement to maintain a specific amount of NA per dose. Yet, there is a large body of data supporting the contribution of anti-NA antibodies to protection from severe influenza disease.^38–41^ It is postulated that induction of NAI titers by a next-generation influenza vaccine could provide better protection against drifted influenza strains. In the FLU-GFPDL analysis, an increase in bound phages containing inserts was observed that mapped to NA, including targets close to the enzymatic site (aa R670, E671, D704, R705, R779, E839 based on HA-NA numbering)^25^ using serum samples from the AS03-adjuvanted vaccine group (Fig. 2b). To further investigate the functional relevance of this observation we tested individual samples from both vaccine groups using the standardized neuraminidase inhibition assay.^42^ As can be seen in Fig. 6, the fold change in NAI titers (D42 vs. D0) was significantly higher for the AS03-adjuvanted vaccine recipients compared with the unadjuvanted vaccine group (*p* = 0.0269).

Together, our study demonstrated profound changes in the antibody epitope repertoire and affinity maturation following vaccination with a novel H5N1 immunogen from avian influenza strain with pandemic potential when combined with the oil-in-water adjuvant AS03. The changes in the quality of the antibody responses correlated with an expanded breadth of neutralization against heterologous clades of H5N1, but not against heterosubtypic Group 1 H1N1 influenza viruses.

## Discussion

In the current study, FLU-GFPDL and SPR analyses were used for comprehensive analysis of the humoral responses generated by the FDA-licensed H5N1 A/Indonesia monovalent inactivated vaccine, with or without the AS03 oil-in-water adjuvant.^8^ For understanding the impact of AS03 adjuvant on vaccination-induced humoral response, samples were collected prevaccination (day 0), 3 weeks post first dose (day 21), 3 weeks post second dose (days 42) and at day 100, for antibody analyses.

The main findings of the study were: (i) the unadjuvanted low dose vaccine (3.75 µg HA/dose) was poorly immunogenic in terms of eliciting neutralization titers, while the AS03-adjuvanted vaccine was strongly immunogenic leading to 100% seroconversion rates by day 42. Significant cross-clade neutralization of other clade 2 H5N1 strains was also found, with a lower response rate against H5N1 clade 1 virus; (ii) GFPDL and SPR demonstrated H5-binding antibodies in prevaccination sera. These antibodies targeted an epitope within the HA2 domain with a high degree of homology (>90%) to the corresponding sequence in the HA2 of group 1 (H1N1) human seasonal viruses. These HA2 binding antibodies were increased after vaccination with the H5N1 vaccine in both unadjuvanted and adjuvanted vaccine groups; (iii) vaccination with AS03-adjuvanted H5N1 vaccine (but not with unadjuvanted vaccine) induced epitope spreading from HA2 to HA1 (including large fragments spanning the RBD) resulting in a sevenfold increase in the ratio of HA1/HA2 bound inserts compared to unadjuvanted vaccination; (iv) SPR real-time kinetic analyses with HA1 and HA2 domains revealed a gradual increase in the total binding and affinity of antibodies targeting the globular HA1 domain primarily in recipients of the AS03-adjuvanted H5N1 vaccine; (v) a strong inverse correlation was found between the anti-HA1-binding antibody off-rates (surrogate of antibody affinity) and the neutralization titers against either A/Indonesia (vaccine strain) or the other clade 2 viruses. In contrast, no clear correlation was found between HA2 binding titers (or off-rates) and virus neutralization titers; (vi) expansion of binding to targets in the neuraminidase protein (NA), including sites close to the sialic acid binding enzymatic site was also observed for sera from AS03-adjuvanted vaccine recipients using GFPDL. In agreement, the increase in neuraminidase inhibition (NAI) titers was more pronounced in this group; (vii) no increase in heterosubtypic neutralization of Group 1 H1N1 seasonal influenza viruses was found in either H5N1 vaccine group.

The response rate against the unadjuvanted H5N1-A/Indonesia vaccine in the current study was lower than that reported against H5N1 A/Vietnam in the earlier vaccine trial that supported licensure of the monovalent inactivated vaccine from Sanofi Pasteur.^43^ It is likely that the main reason for this difference is the vaccine dose. The unadjuvanted H5N1-A/Vietnam vaccine contained a 90 µg HA/dose, which is sixfold higher than the antigen dose in seasonal vaccine (15 µg HA) and 24-fold higher than the antigen dose of H5N1-A/Indonesia (3.75 µg HA) that was approved by the FDA in combination with AS03 used in the current study. Therefore, the addition of AS03 provided a significant dose-sparing effect that will be of great value in the case of mass global vaccination. Similar to the finding with H5N1 immunogens, several clinical trials conducted recently demonstrated improved immunogenicity and dose sparing of H7N9 vaccines when combined with AS03^44,45^ and with MF59.^45^

Since the HA2 sequence is conserved among Group 1 H5N1 and H1N1 strains, it has been hypothesized that vaccination with H5N1 in adult populations that have been exposed to seasonal influenza strains, either due to seasonal influenza infection or vaccination will recall HA2 stem-specific antibodies that can cross-protective Group 1 H1N1 strains; this is the basis for development of stem-based next-generation influenza vaccines. It is postulated that adding an adjuvant like AS03 will significantly enhance the magnitude of such HA2-specific recall response early after vaccination with novel head containing vaccines such as those for H5N1. Indeed, we found an increase in HA2 binding antibodies on day 21 post first vaccination using both GFPDL and SPR. Therefore, we evaluated the possibility that soon after vaccination broad heterosubtypic antibodies could be detected in the sera specific for the more conserved HA2 stem domain, by measuring neutralization of H1N1 strains that circulated in the US a decade ago. A modified neutralization assay (72 h) with increased sensitivity to detect stem-targeting broadly neutralizing antibodies was used.^46^ The H1N1 strains used included A/New Caledonia/1999, A/ Solomon Islands/2006, and A/Brisbane/59/2007, which circulated in the US in the years 1999–2005, 2006/7, and 2007–2009, respectively. Since H5 and H1 globular heads share low homology, any increase in anti-H1N1 neutralization titers between day 0 and day 7 post vaccination should reflect a recall of HA2 stem-targeting neutralizing antibodies. However, we found no clear increase in neutralization titers against the three H1N1 strains, in spite of low prevaccination titers against these seasonal H1N1 influenza strains. Therefore, the AS03-adjuvanted H5N1 vaccine used in our study (containing a novel HA1 head domain) did not fulfill the requirements for “A Universal Influenza Vaccine” as outlined in a recent white paper.^32^ One of the limitations to this conclusion is the fact that currently there is no established correlate of protection for stem-based influenza vaccines. In a previous study, passive transfer of antibodies from H5N1-vaccinated individuals (inactivated influenza virosomes from vaccine strain RG14 containing HA from H5N1 A/Vietnam/1194/2004 reassorted with H1N1 PR8 internal genes, NIBSC, UK at 30 μg HA/dose) protected mice from challenge with a chimeric H9/1 N3 strain.^33^ It was postulated that Fc-mediated functions play a key role in such protection.^47^ Multiple in vitro and in vivo assays based on the antibody Fc functions have been described including antibody-dependent cellular cytotoxicity (ADCC), antibody-dependent cellular phagocytosis, and antibody-dependent cell-associated viral elimination. To date, no one assay has been fully predictive of in vivo protection against heterotypic challenge viruses in humans and animal models.^48,32,34,47,49–55^ Furthermore, the potential of non-HAI stem-targeting antibodies to enhance lung pathology after infection, in absence of head-targeting antibodies, should be carefully evaluated in preclinical models.^56–59^

Previous studies in humans using AS03-adjuvanted H1N1pdm09 vaccine combined with B-cell receptor (BCR) sequencing of isolated of plasma blasts on day 7 provided evidence for improved recruitment of total antibody secreting cells, that included naïve (unmutated BCR) B cells and recalled preexisting memory B cells in the adjuvanted group compared to the unadjuvanted group.^60^ In a separate study, subjects receiving AS03-adjuvanted H1N1pdm09^46^ showed an increase in both anti-head and anti-stem antibodies. A significant increase in HI titers against the homologous strain was found in both previously vaccinated (with a seasonal influenza vaccine) or unvaccinated individuals (average fold increase of 32 and 250, respectively). In contrast, a very modest increase in neutralization of a chimeric virus expressing cH9/1N1 and ADCC (using cH6/1 HA-coated plates and CD16^+^ NK cell line) (<2 fold) was reported.^46^ Therefore, the conclusions of that study regarding heterosubtypic immunity are not significantly different from our study using AS03-adjuvanted avian influenza strain (H5N1 A/Indonesia) vaccine.

The NA gene in seasonal and avian influenza was shown to undergo differential evolution with fewer seasonal drifts compared to the HA gene.^61^ It has been suggested that NA should be a component of next-generation influenza vaccines since NA-inhibiting antibodies were shown to correlate with protection from severe influenza disease.^41,55^ In the current study, AS03-adjuvanted H5N1 vaccine promoted an expansion of the anti-NA epitope repertoire in serum antibodies (Fig. 2) along with corresponding significant increase in the NAI titers (Fig. 6). Therefore, adjuvanted NA-based vaccines are likely to elicit even stronger cross-reactive NAI titers.

These data demonstrate both strong antibody responses with high affinity and epitope spreading of antibody repertoire following vaccination with AS03-adjuvanted (but not unadjuvanted) H5N1 vaccine, suggesting recruitment of naïve B cells with BCRs targeting important protective sites in the HA1 globular domain and the NA proteins contained in the H5N1 vaccine. Future influenza vaccines that incorporate both HA and NA components are likely to benefit from oil-in-water adjuvants such as AS03 and similar platforms.

While adjuvants in combination with traditional influenza vaccines (inactivated, split or subunit) have not yet provided the breadth and duration of protection required from a universal influenza vaccine, certain adjuvants most likely will be required for optimal and sustainable immune responses against the next-generation influenza vaccines. Thus, an iterative approach for selection of the most promising immunogen design, and the optimal adjuvant will be an important component in the development of this new generation of influenza vaccines.

## Materials and methods

### Serum samples

Serum samples from H5N1 human vaccine trial were obtained from the Center for Human Immunology (CHI)-sponsored A/Indonesia/5/05 vaccine trial using the FDA-approved AS03-adjuvanted H5N1 (A/Indonesia/05/2005) vaccine conducted at NIAID, NIH. The study protocol was approved by the NIH Institutional Review Board and the FDA (ClinicalTrials.gov Identifier: NCT01578317). A written informed consent was obtained from all participants. All samples were de-identified. The study samples were coded and all the antibody assays were performed blindly. After the data were generated, samples were unblinded to perform the data analysis in this study. The protocols were evaluated by CBER/NIH Research Involving Human Subjects Committee and were conducted under RIHSC exemption #03-118B.

### Construction of H5N1 (A/Indonesia/5/2005) HA/NA gene-fragment phage display libraries

The phage display libraries expressing inserts spanning the HA and NA genes of the H5N1-A/Indonesia/05/2005 strains were constructed as previously described for H5N1 A/Vietnam/1203/04 GFPDL.^20,21^ fSK-9-3 is a gIIIp display-based phage vector where the desired polypeptide can be expressed as a gIIIp fusion protein. The phage display libraries used in the current study expressed inserts spanning the HA and NA genes (referred as HA-NA).

### Panning of H5N1 GFPD libraries with polyclonal human vaccine sera

For each round of panning, an equal volume of sera was used for each group. Prior to panning of GFPDL with polyclonal human antibodies, serum components, which might nonspecifically interact with phage proteins, were removed by incubation with ultra violet-killed M13K07 phage-coated Petri dishes. Subsequent GFPDL selection was carried out in solution (with Protein A/G-Sepharose) and inserts of bound phages were PCR amplified and sequenced as previously described.^20,21^

### Generation of H5N1-HA1 recombinant proteins

The DNA gene segments corresponding to the HA1 and HA2 proteins of H5N1-A/Indonesia/05/2005 and H5N1-A/Vietnam/1203/2004 were cloned as *Not*I-*Pac*I inserts into a T7 promoter-based pSK expression vector in which the desired polypeptide can be expressed as a fusion protein with His_6_ tag at the C-terminus. The HA1 and HA2 proteins were expressed and purified as described before.^23^ Briefly, *E. coli* Rosetta Gami cells (Novagen) were used for expression of H5N1 proteins. Following expression, inclusion bodies were isolated by cell lysis and multiple washing steps, denatured, refolding in redox folding buffer and dialyzed. The dialysate was filtered through a 0.45 µm filter and was subjected to purification by HisTrap Fast flow chromatography. The purified proteins were characterized for presence of oligomers by Gel filtration chromatography and functional binding to RBC in a hemagglutination assay.

### Affinity measurements by surface plasmon resonance (SPR)

Steady-state equilibrium binding of post-H5N1-vaccinated human sera was monitored at 25 °C using a ProteOn surface plasmon resonance biosensor (Bio-Rad).^22^ The recombinant HA globular domain (rHA1-His_6_) or HA stalk domain (rHA2-His_6_) for the A/Indonesia/05/2005 (clade 2.1) or from H5N1- A/Vietnam/1203/2004 influenza virus strain was coupled to a GLC sensor chip with amine coupling with 100 and 500 resonance units (RU) in the test flow cells. Samples of 60 µL sera at 10-fold and 100-fold dilutions were injected at a flow rate of 50 µL/min (120-s contact time) for association, and disassociation was performed over a 600-s interval (at a flow rate of 50 µL/min). Responses from the protein surface were corrected for the response from a mock (no coating) surface and for responses from a separate, buffer only injection. Binding kinetics for the human vaccine sera and the data analysis were calculated with Bio-Rad ProteOn manager software (version 3.0.1). Antibody off-rate constants, which describe the fraction of antigen−antibody complexes that decay per second, were determined directly from the serum/plasma sample interaction with rHA1 or rHA2 protein using SPR in the dissociation phase and calculated using the Bio-Rad ProteOn manager software for the heterogeneous sample model as described before.^22^ Off-rate constants were determined from two independent SPR runs.

### H5N1 neutralization assay

Viral-neutralizing activity was analyzed in an MN assay in MDCK cells based on the methods of the pandemic influenza reference laboratories of the Centers for Disease Control and Prevention (CDC) with minor modifications provided in the updated SOP issued by the CDC. Antibody-neutralization titers by MN were measured against H5N1 vaccine strains of A/Vietnam/1194/2004 (clade 1), A/Indonesia/5/2005 (clade 2.1), A/Turkey/15/2006 (clade 2.2), A/Egypt/3072/2010 (clade 2.2), and A/Anhui/1/2005 (clade 2.3.4) as well as the H1N1pdm09 (A/California/7/2009). Sera were tested at an initial dilution of 1:20, and those that were negative (<1:20) were assigned a titer of 10. All sera were tested in triplicate, and the geometric mean value was used for analysis.

### Modified neutralization assay for seasonal influenza strains

For measuring cross-subtypic neutralization activity against H1N1 and H3N2 seasonal influenza strains that is more sensitive to neutralizing activity due to stem-specific antibodies, a 3-day infection assay was performed.^34^ MDCK cells (2×10^5^/100 µL/well) were seeded in flat bottom 96-well plates and incubated at 37 °C overnight. The following day, twofold serially diluted RDE-treated serum samples (starting at 1:10) were added to a separate 96-well tissue culture plate in duplicates. Diluted sera were incubated with 100 TCID_50_/100 µL/well influenza virus (H1N1; A/New Caledonia/22/99, A/Solomon Islands/3/06, A/Brisbane/59/07, A/California/07/2009, or H3N2; A/Switzerland/9715293/2013) for 1 h at room temperature with shaking. Cell-containing 96-well plates were washed with PBS, and 200 µL/well of the virus-sera mixture was added and incubated at 37 °C for 72 h. Plates were centrifuged at 3000 rpm for 5 min and 50 µL of the supernatant was added to 50 µL of 1% human red blood cells in a U-bottom 96-well plate. Agglutination was read after incubation for 60 min at 37 °C.

### Neuraminidase inhibition (NAI) assay

The enzyme-linked lectin assay (ELLA) was used for the detection of NA-inhibiting antibodies in immune antisera as previously described.^42^ To prevent false NAI signals due to interfering HA antibodies, virus reassortants that combine H5N1 NA and a mismatched HA of the H6 subtype was generated by reverse genetics as previously described^62^ to measure H5N1-specific NA activity inhibiting antibody titers.

### Statistical analyses

Differences between groups (*p* values) were examined for statistical significance by the multiple comparison adjustment using the Bonferroni method. In addition to *p* values, the 95% confidence intervals of the differences between study groups are also provided. A *p* value less than 0.05 was considered significant. Analysis was conducted without alpha adjustment for multiplicity. Spearman correlations are reported for the calculation of correlations between off-rate and MN titers combined across all vaccine groups. All statistical calculations were performed using ANOVA.

### Data availability

The data sets generated during and/or analyzed during the study are available from the corresponding author upon reasonable request.

## Electronic supplementary material

Supplementary Figure 1

## Electronic supplementary material

**Supplementary information** accompanies the paper on the *npj Vaccines* website (10.1038/s41541-018-0076-2).
