# Supplementary figures and images for: AS03-adjuvanted H5N1 vaccine promotes antibody diversity and affinity maturation, NAI titers, cross-clade H5N1 neutralization, but not H1N1 cross-subtype neutralization
Source: NPJ Vaccines. 2018 Oct 1;3:40. doi: 10.1038/s41541-018-0076-2 (PMC6167326; doi:10.1038/s41541-018-0076-2)

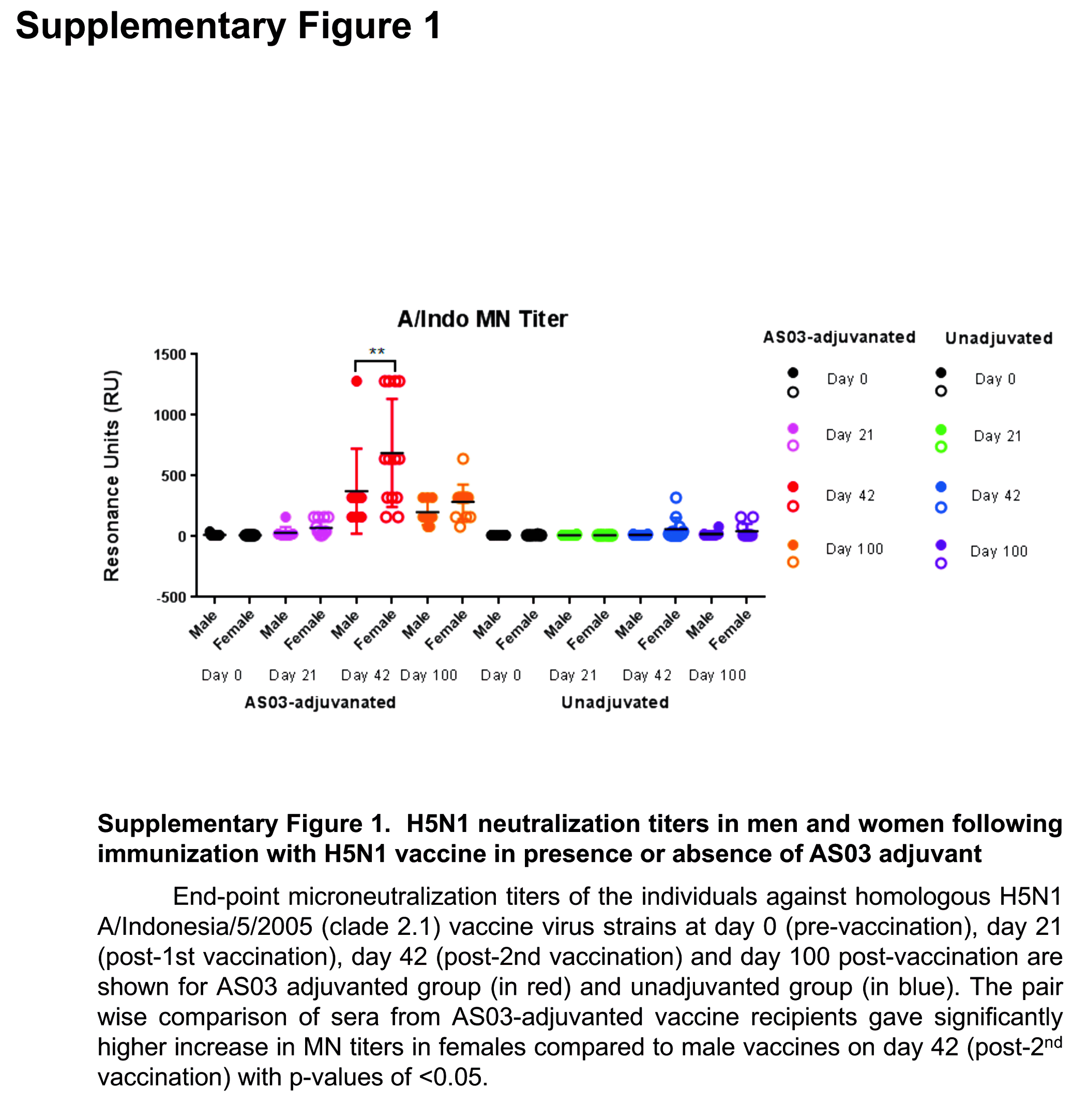

Supplement: Supplementary file 1 — Supplementary Figure 1 [file 41541_2018_76_MOESM1_ESM.tif]
